# P4HA2 involved in SLUG-associated EMT predicts poor prognosis of patients with KRAS-positive colorectal cancer

**DOI:** 10.1007/s00795-024-00385-0

**Published:** 2024-03-24

**Authors:** Heba El-Deek Mohammed El-Deek, Maha Salah El-Naggar, Aiat Morsy Mohamed Morsy, Mayada Fawzy Sedik, Heba Ahmed Osman, Asmaa M. Ahmed

**Affiliations:** 1 Department of Pathology, Faculty of Medicine, Assiut University, Assiut, Egypt; 2 Department of Clinical Oncology, Faculty of Medicine, Assiut University Hospital, Assiut, Egypt; 3 Department of Medical Oncology and Hematological Malignancies, South Egypt Cancer Institute, Assiut University, Assiut, Egypt; 4 Department of Tropical Medicine and Gastroenterology, Faculty of Medicine, South Valley University, Qena, Egypt

**Keywords:** SLUG, P4HA2, CRC

## Abstract

This study aimed to examine the immunohistochemical expression of epithelial–mesenchymal transition biomarkers: P4HA2 and SLUG in colorectal carcinoma (CRC) specimens, then to assess their relation to clinicopathological features including KRAS mutations and patients’ survival, and finally to study the correlation between them in CRC. The result of this study showed that SLUG and P4HA2 were significantly higher in association with adverse prognostic factors: presence of lympho-vascular invasion, perineural invasion, higher tumor budding, tumor stage, presence of lymph node metastasis, and presence of distant metastasis. CRC specimens with KRAS mutation were associated with significant higher SLUG and P4HA2 expression. High expression of both SLUG and P4HA2 was significantly unfavorable prognostic indicator as regards overall survival (OS) and disease-free survival (DFS). In KRAS mutated cases, high P4HA2 expression was the only significant poor prognostic indicator as regarding DFS. In conclusions, our data highlight that both SLUG and P4HA2 expression may serve as potentially important poor prognostic biomarkers in CRC and targeting these molecules may be providing a novel therapeutic strategy. In KRAS mutation group, high P4HA2 expression is the only independent prognostic factor for tumor recurrence, so it can be suggested to be a novel target for therapy.

## Introduction

Colorectal cancer (CRC) is among the most prevalent malignant tumors and one of the leading causes of cancer-related death worldwide. Despite the great advance in the diagnosis and development of therapeutic options, a significant number of patients unfortunately develop local advanced disease or even distant metastasis [1]. In particular, CRC patients with mutated KRAS are challenging to treat as KRAS mutation drives more aggressive behavior, metastatic potential, and even therapeutic resistance to target therapy [2]. A better understanding of different biological events contributing to CRC carcinogenesis and aggressiveness may be necessary for better stratification of the risk of recurrence, and the development of novel targets for diagnosis and therapy.

Among the pathogenic mechanisms of carcinogenesis, epithelial–mesenchymal transition (EMT) has gained much attention. EMT is a process whereby epithelial cells lose their epithelial characteristics and acquire a mesenchymal phenotype, with decreased adhesion and enhanced migration or invasion of different cancers including CRC [3, 4]. Increasing evidences propose that several transcriptional proteins that are involved in collagen biosynthesis and deposition, such as collagen prolyl-4-hydroxylase, can promote cancer progression through the regulation of EMT [3].

There are three subtypes of collagen prolyl-4-hydroxylases α isoforms (P4HA1, P4HA2, and P4HA3) [5]. P4HA2 is one of the regulator enzymes involved in the remodeling of the extracellular matrix (ECM) and collagen modification [6]. Recently, dysregulation of P4HA2 has been implicated in various types of cancers, such as oral squamous cell carcinoma [7], breast cancer [8], hepatocellular carcinoma [9], and pancreatic cancer [10]. Until now, controversy exists regarding its prognostic role; while its lower expression was associated with low survival in pancreatic carcinoma [10], its higher level was significantly associated with a shorter overall survival in hepatocellular carcinoma [9], and breast cancer [8]. To date, P4HA2 expression pattern, biological functions, and its oncogenic role in CRC have not been previously investigated, specifically in CRC with KRAS mutation.

Another transcriptional factor that plays a crucial role in the regulation of EMT is the SLUG protein. It is a member of the Snail family of zinc finger transcription factors that have been shown to participate in mesoderm formation [11]. During cancer progression, SLUG could lead to increased motility, invasiveness, and metastatic capabilities of cancer cells [12, 13]. This is achieved by the ability of SLUG to bind to E-boxes of E-cadherin promoter and repress E-cadherin expression leading to the acquisition of invasive and migratory properties [14].

Some studies showed that high levels of SLUG expression were correlated with disease relapse and decreased survival in breast, esophageal, and colorectal carcinoma [15, 16]. Therefore, SLUG expression might be an important prognostic parameter of many cancers including colorectal cancer.

Interestingly, Lin, Jiang et al. demonstrated a positive correlation between P4HA2 and SLUG in glioma [6]. However, the correlation between these proteins hasn’t been previously investigated in CRC.

To date, the knowledge about the expression pattern of P4HA2 and SLUG in CRC and their relation to different prognostic parameters including KRAS mutation is limited. This study aims to examine the immunohistochemical expression pattern of both P4HA2 and SLUG in CRC specimens, then to assess their relation to different clinicopathological features including KRAS mutations and patients’ survival, and finally to study the correlation between both proteins in CRC.

## Materials and methods

### Specimens

This is a retrospective study included 70 specimens of colorectal carcinoma. These were retrieved from the archive of the Surgical Pathology Laboratory, Assiut University Hospital, Faculty of Medicine, Assiut University and from archives of Pathology Department in South Egypt Cancer Institute, Assiut University (between years Jan 2016–Jan 2022).

The available clinicopathological data of the cases were obtained from the hospital medical records at Pathology Department, Clinical Oncology Department, Assiut University Hospital, Faculty of Medicine, Assiut University. Some data were also obtained from South Egypt Cancer Institute, Assiut University and Tropical Medicine and Gastroenterology Department, Qena University, Faculty of Medicine. These data include: age and sex of the patient, tumor site, operation type, KRAS mutation, clinical follow-up information as the occurrence of distant metastasis or local recurrence, and survival data including overall survival (OS) and disease-free survival (DFS).

Representative hematoxylin and eosin-stained slides of tumors were examined for each specimen for identification of the following features; histologic type and grade (according to WHO (World Health Organization) classification of colon and rectal tumors, 5th Edition, 2019) [17], lympho-vascular (LVI) and perineural invasion (PNI), depth of tumor invasion (T), lymph node metastasis (N), and distant metastasis (M) (according to the TNM classification of the American Joint Committee on Cancer (AJCC) 8th Edition, 2017) [18]. In addition, we evaluate tumor budding and the presence of poorly differentiated clusters (according to The International Tumor Budding Consensus Conference (ITBCC) 2016 group) [19].

This study was approved by the institutional ethics and research committee, Faculty of Medicine, Assiut University (IRB number 3000014).

### Immunohistochemical staining

Immunohistochemical staining was performed using avid-biotin immunoperoxidase method. Four µm thickness of formalin-fixed paraffin-embedded specimens was taken from tissue blocks and mounted over coated slides. Sections were dewaxed and rehydrated through graded alcohols to distilled water. The hydrogen peroxide block was applied and incubated for 15 min then for antigen retrieval; sections were treated in the microwave of (600 watts) by immersion of the slides in citrate buffer solution (PH 7) for 20 min. Sections were incubated with primary antibodies. The antibodies used were SLUG (clone OTI1G7, Thermo Scientific, diluted at 1/150) and P4HA2 (clone CL0351, Thermo Scientific, diluted at 1/100) for 1 h at room temperature in the humid chamber. Then, the slides were washed 2–3 times using phosphate-buffered saline solution. After washing, secondary staining kits were used according to the manufacturer’s instructions (Thermo Scientific, Fremont, CA, USA). Counterstaining was done using Mayer’s hematoxylin and examined by light microscopy.

### Evaluation of SLUG and P4HA2

Both SLUG and P4HA2 were assessed semiquantitatively using IHC (H score); (IHC score was calculated by multiplying the degree of staining “0, 1, 2, 3” by the percentage of positive staining (“0–100%”) to give a maximum histoscore of 300 [20, 21]. The final H score was calculated as follows: H score = {(0 × % negative cells) + (1 × % weak positive cells) + (2 × % moderate positive cells) + (3% strong positive cells)} with the overall score ranging from 0 (negative) to 300 (100% strong staining). The histoscore was independently viewed and scored by two pathologists without disclosing the clinical data of these patients.

For survival analysis, the expression of both proteins was divided into low and high expression according to the median which was 210 for both SLUG and P4HA2 proteins.

### Statistical analysis

All statistical calculations were done using SPSS (statistical package for the social science; SPSS Inc., Chicago, IL, USA) version 22. Mann–Whitney test and Kruskal–Wallis (*K*-test) were used to compare the means of SLUG and P4HA2 expression in the studied cases in relation to different clinicopathological features. Spearman correlation coefficient was used to investigate the correlation between the two markers. The prognostic effect of the various parameters on clinical outcome was tested using the Kaplan–Meier method with the log-rank test was applied to compare survival curves. Multivariate analysis was done using the Cox regression model. *p* values of < 0.05 were regarded as statistically significant.

## Results

### Clinicopathological characteristics

The clinicopathological characteristics are summarized in (Table 1). Briefly, 70 CRC patients were included in the study (40 females and 30 males). The mean age at the time of diagnosis was 45.47 ± 13.68 (range 19–80 years). All cases were adenocarcinoma, with 12 (17.1%) cases were grade 1, 49 (70%) cases were grade 2 and 9 (12.9%) cases were grade 3 (Table 1).

**Table 1:** Clinicopathological characteristics

Clinicopathological characteristics	No. (70)	%
**Sex**		
Male	30	42.90
Female	40	57.10
**Age (years)**		
≤ 50	47	67.10
> 50	23	32.90
Mean ± SD (range)	45.47 ± 13.68 (19–80)	
**Grade and differentiation**		
Well differentiated (G1)	12	17.10
Moderate differentiation (G2)	49	70
Poorly differentiated (G3)	9	12.90
**Lymph–vascular invasion (LVI)**		
Positive	18	25.70
Negative	52	74.30
**Peri-neural invasion(PNI)**		
Positive	10	14.50
Negative	59	84.50
**Tumor budding**		
1	22	31.40
2	33	47.10
3	15	15
**Poorly differentiated clusters**		
1	23	32.90
2	32	45.70
3	15	21.40
**T stage**		
T2	30	42.9
T3	25	35.70
T4	15	21.40
**Lymph node metastasis**		
N0	34	48.60
N1	14	20
N2	22	31.40
**Distant metastasis**		
M0	39	55.70
M1	31	44.30
**KRAS**		
Wild	45	64.30
Mutant	25	35.70
**Local recurrence**		
Yes	16	22.90
No	54	77.10

### Immunohistochemical expression of SLUG and P4HA2

**Fig. 1:**
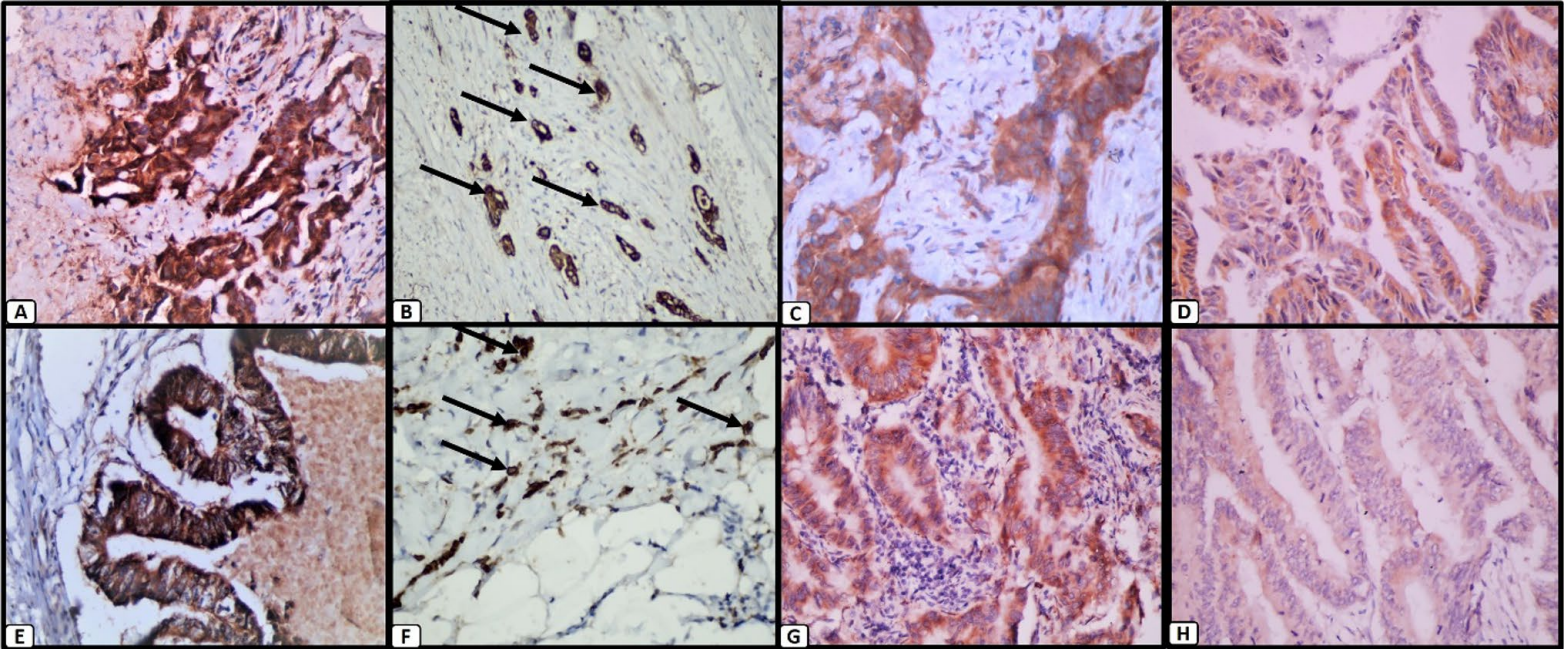
Expression of P4HA2 and SLUG in colorectal carcinoma. Colorectal carcinoma tissue showing; **A** strong cytoplasmic expression of P4HA2 (×400), **B** strong cytoplasmic expression of P4HA2 in areas of high tumor budding (×400), **C** moderate cytoplasmic expression of P4HA2 (×400), **D** mild cytoplasmic expression of P4HA2 (×400), **E** strong cytoplasmic expression of SLUG (×400), **F** strong cytoplasmic expression of SLUG in areas of high tumor budding (×400), **G** moderate cytoplasmic expression of SLUG (×400), and **H** mild cytoplasmic expression of SLUG (×400)

SLUG and P4HA2 were investigated in 70 CRC specimens. Both markers were observed mainly in the cytoplasm of tumor cells. Positive staining of SLUG and P4HA2 was detected in 65/70 (95%) and 67/70 (95.7%) CRC specimens respectively (Fig. 1).

### Relationship between SLUG and P4HA2 expression and clinicopathological criteria

The mean cytoplasmic expression of SLUG and P4HA2 was significantly higher in association with adverse prognostic factors: presence of LVI (*p* = 0.027 and *p* = 0.022) respectively, presence of PNI (P = 0.010and 0.016) respectively, higher tumor budding (*p* = 0.006 and *p* < 0.0001) respectively, higher T stage (*p* < 0.0001) for each, presence of LN metastasis (*p* < 0.0001) for each, and presence of distant metastasis (*p* < 0.0001) for each. In addition, CRC specimens that showed KRAS mutation were associated with significant higher SLUG and P4HA2 expression (*p* = 0.007 and *p* = 0.002) respectively (Table 2).

**Table 2:** Relationship between SLUG and P4HA2 expression and clinicopathological features

Clinicopathological characteristics	SLUG expression		P4HA4 expression	
	Means ± SD	*p* value	Means ± SD	*p* value
**Sex**				
Male	165.83 ± 98.23	0.392	170.17 ± 92.88	0.369
Female	187 ± 90.64		190.38 ± 89.76	
**Age (years)**				
≤ 50	192.55 ± 95.89	0.051	194.79 ± 92.63	0.052
> 50	149.57 ± 84.50		155.55 ± 83.20	
**Grade and differentiation**				
Well differentiated (G1)	140.83 ± 75.64	0.114	149.17 ± 72.76	0.242
Moderate differentiation (G2)	179.90 ± 98.55		183.67 ± 93.98	
Poorly differentiated (G3)	220.56 ± 76.17		214.44 ± 91.29	
**Lymph–vascular invasion (LVI)**				
Positive	218.61 ± 89.74	0.027	221.39 ± 89.44	0.022
Negative	164.52 ± 92.08		167.98 ± 88.26	
**Peri-neural invasion (PNI)**				
Positive	245.00 ± 74.38	0.010	240.00 ± 88.34	0.016
Negative	168.47 ± 93.08		172.88 ± 88.99	
**Tumor budding**				
1	107.73 ± 92.73	0.006	111.82 ± 87.852	
2	192.27 ± 77.98		191.82 ± 73.25	< 0.0001
3	251.67 ± 51.18		262.00 ± 46.36	
**Poorly differentiated clusters**				
1	177.17 ± 94.66	0.690	176.96 ± 96.71	0.663
2	184.69 ± 99.04		189.06 ± 93.04	
3	167.00 ± 86.24		173.33 ± 81.93	
**T stage**				
T2	121.67 ± 85.48	< 0.0001	126.50 ± 79.76	< 0.0001
T3	191.00 ± 80.99		191.40 ± 80.11	
T4	271.00 ± 26.87		276.00 ± 20.28	
**Lymph node metastasis**				
N0	192.06 ± 81.80	< 0.0001	136.91 ± 78.11	< 0.0001
N1	196.43 ± 80.51		196.07 ± 72.14	
N2	238.64 ± 83.41		241.82 ± 85.02	
**Distant metastasis**				
M0	116.97 ± 77.87	< 0.0001	120.00 ± 73.67	< 0.0001
M1	255.97 ± 38.13		259.35 ± 31.51	
**KRAS**				
Wild	155.33 ± 93.62	0.007	156.11 ± 89.64	0.002
Mutant	220.00 ± 80.55		227.80 ± 75.07	

No statistically significant difference was detected between the mean of SLUG and P4HA2 expression regarding patient age (*p* = 0.051and *p* = 0.052) respectively, gender (*p* = 0.392 and *p* = 0.369) respectively, tumor grade (*p* = 0.114 and *p* = 0.242), respectively, and the grades of poorly differentiated clusters (*p* = 0.690 and *p* = 0.663) respectively (Table 2).

### Correlation between SLUG andP4HA2 expression in CRC specimens:

A significant strong positive correlation was detected between the expression of SLUG and P4HA2 (r = 0.971, *p* < 0.0001) in CRC (Table 3).

**Table 3:** Spearman correlation coefficient:

		Slug	P4HA2
**Spearman's rho**			
Slug	Correlation coefficient	1.000	0.971
	Sig. (2-tailed)	0.0	0.000
	*N*	70	70
P4HA2	Correlation coefficient	0.971	1.000
	Sig. (2-tailed)	0.000	0.0
	*N*	70	70

### Survival analysis

Survival analysis was carried out following data dichotomization according to the median. The median expression was 210 for both SLUG and P4HA2 markers. Forty (57.1%) specimens showed low SLUG expression while 30 (42.9%) showed high expression. Thirty-seven (52.9%) specimens showed low P4HA2 expression while 33(47.1%) specimens showed high expression.

The mean was 27.97 ± 19.09 (range 1–67 months) for DFS, and 32.67 ± 18.14 (range 2–72 months) for overall survival (OS).

**Fig. 2:**
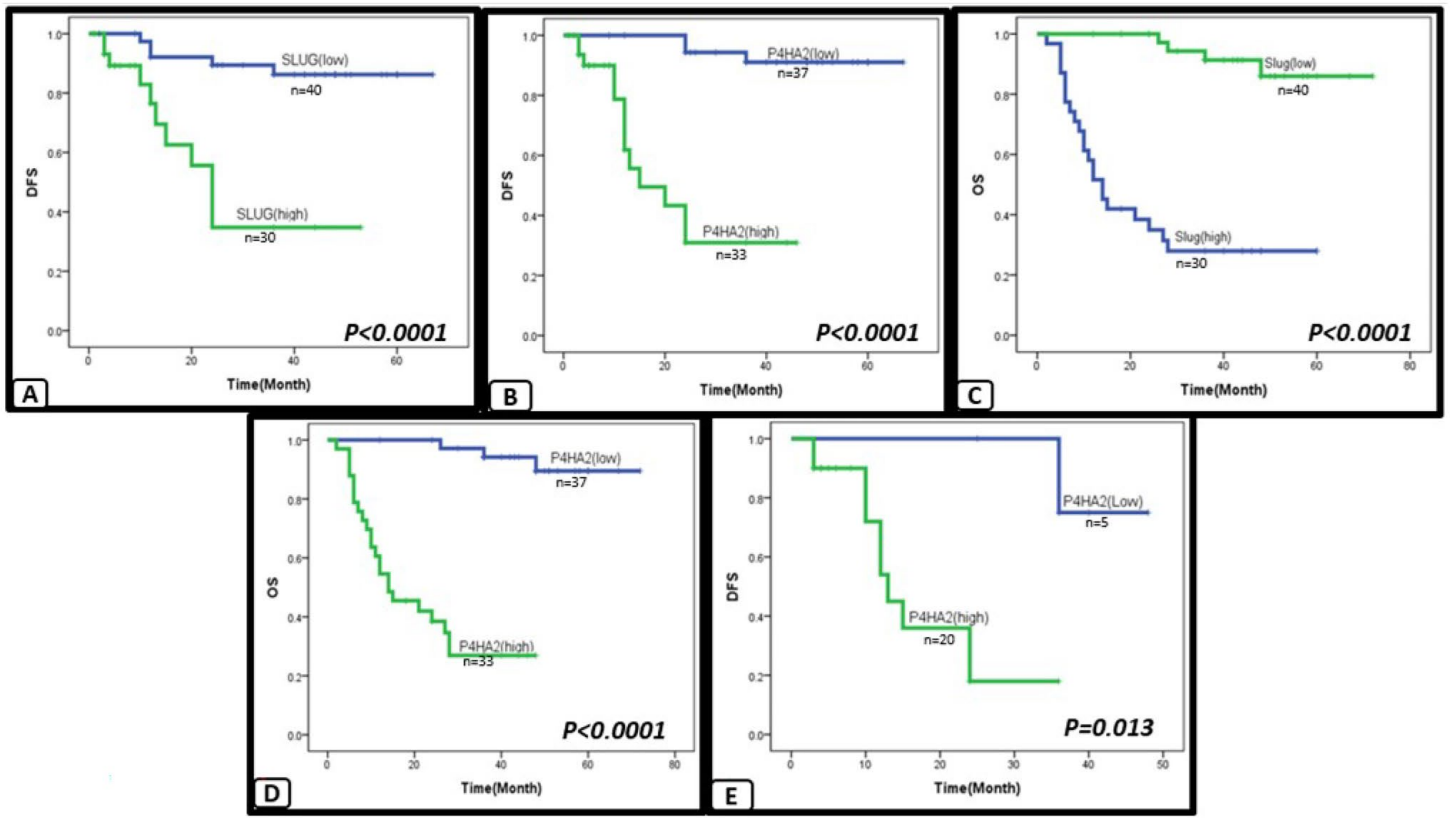
Kaplan–Meier survival curves: the correlation between expression of P4HA2 and SLUG and CRC prognosis, assessed by univariate survival analysis. **A**, **C** high SLUG expression is associated with poor prognosis; disease-free survival (**A**) and overall survival (**C**). **B**, **D** High P4HA2 expression is associated with poor prognosis; disease-free survival (**B**) and overall survival (**D**). **E** High P4HA2 expression is associated with poor disease-free survival in KRAS mutated CRC

Univariate Kaplan–Meier survival analysis demonstrated that high cytoplasmic expression of SLUG and P4HA2 was an unfavorable prognostic indicator as regards overall (OS) and disease-free survival (DFS). The difference achieved statistical significance (SLUG; OS, *p* < 0.0001and DFS; *P* < 0. 0001), (P4HA2; OS, *p* < 0. 0001 and DFS, *p* < 0. 0001) (Fig. 2). In addition, patients with both SLUG high and P4HA2 high showed significant lower OS (*p* < 0. 0001) and DFS (*p* < 0. 0001) by univariate Kaplan–Meier survival analysis.

**Fig. 3:**
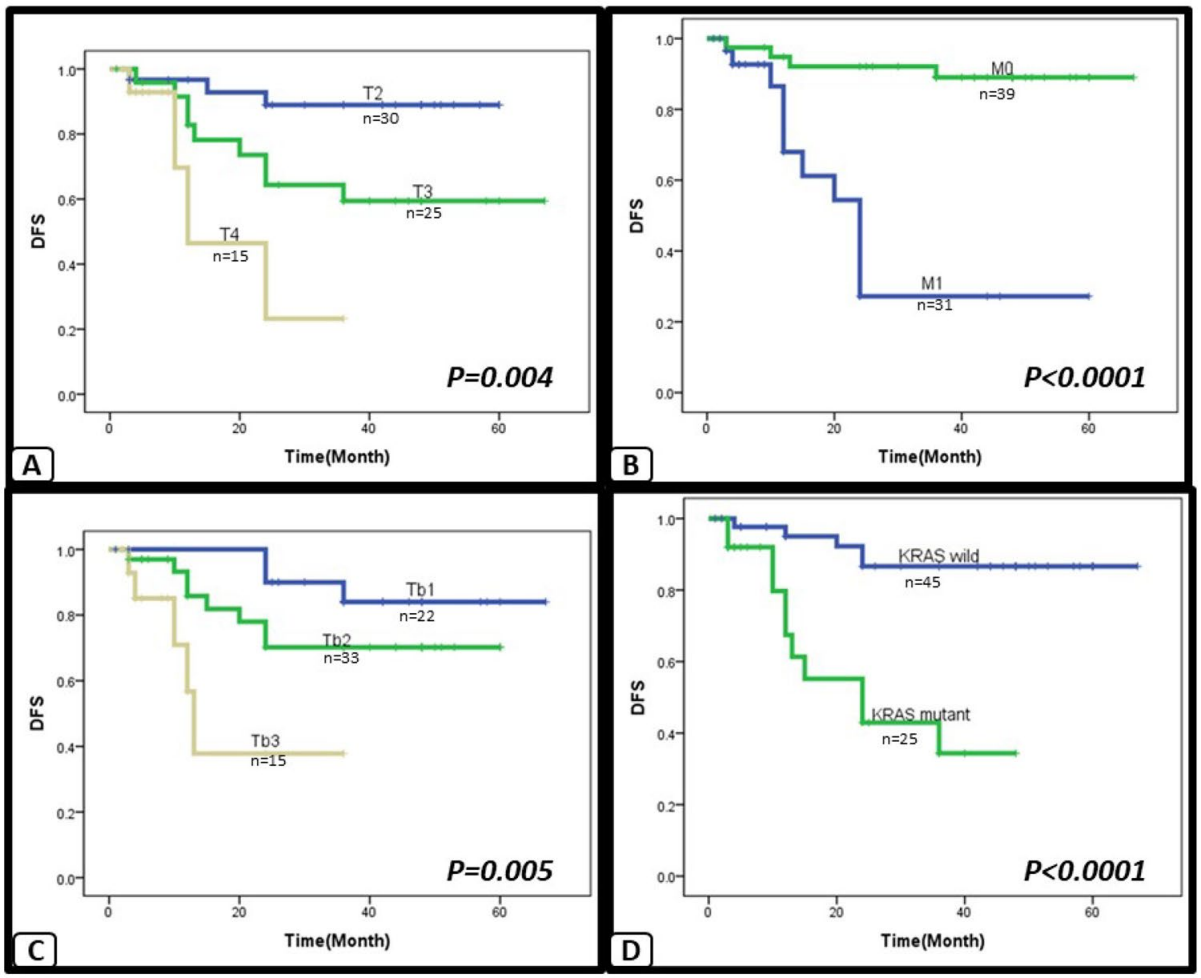
Kaplan–Meier survival curve: the correlation between clinicopathological factors and DFS in CRC, assessed by univariate survival analysis. A progressive decline in DFS is associated with advanced T stage (**A**) presence of distant metastasis (**B**) presence of higher tumor budding (**C**), and presence of KRAS mutations (**D**)

**Fig. 4:**
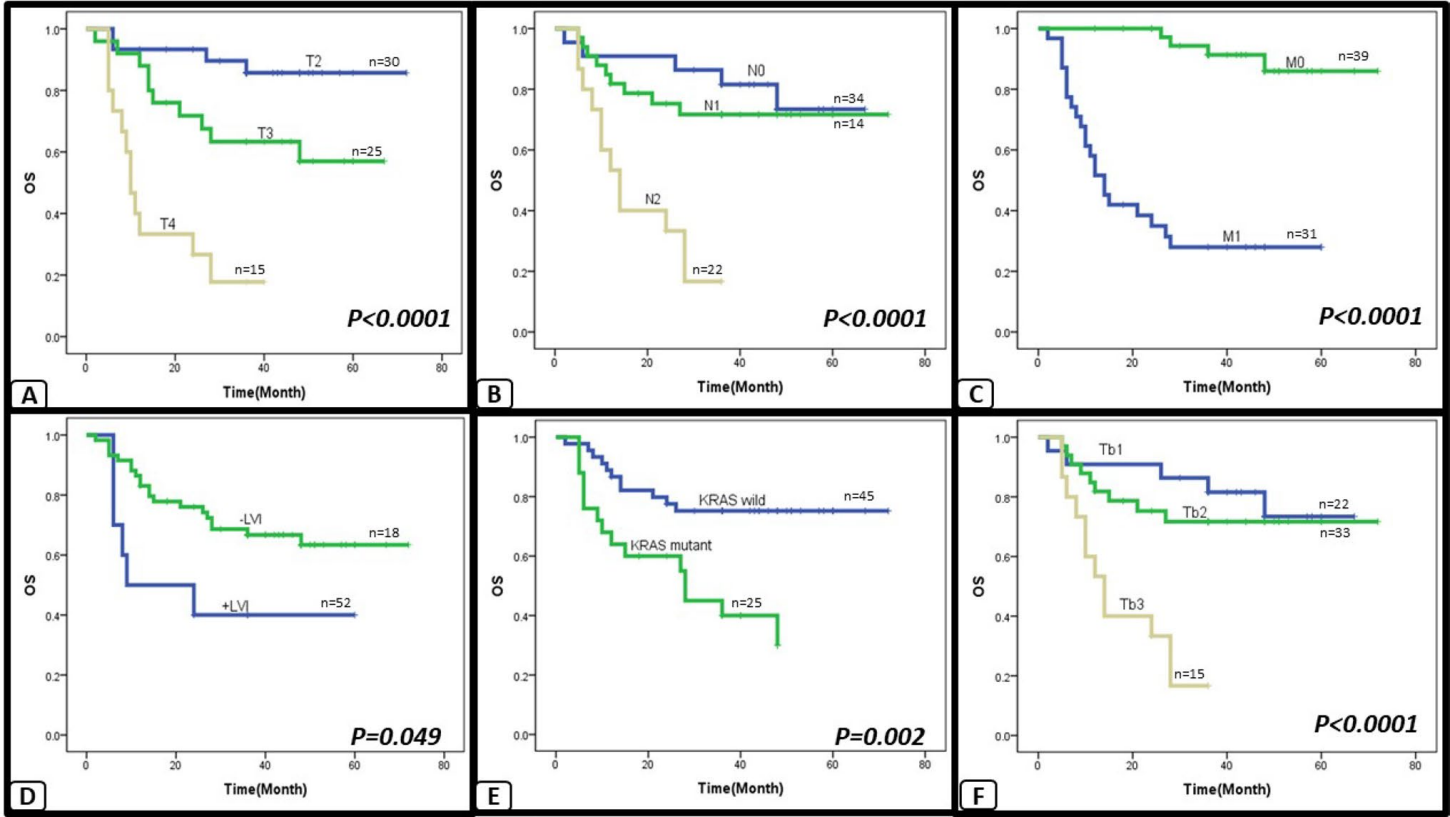
Kaplan–Meier survival curve: the correlation between clinicopathological factors and OS in CRC, assessed by univariate survival analysis. A progressive decline in OS is associated with advanced T stage (**A**), presence of LN metastasis (**B**), presence of distant metastasis (**C**), presence of LVI (**D**), presence of KRAS mutations (**E**) and presence of higher tumor budding (**F**)

The univariate analysis of the other parameters examined showed that there was a progressive decline in both OS and DFS with advanced T stage (OS, *p* < 0.0001 and, DFS, *p* = 0.004) presence of distant metastasis (OS, *p* < 0.0001 and DFS, *p* < 0.0001), presence of higher tumor budding (OS, *p* < 0.0001 and DFS, *p* = 0.005), and with presence of KRAS mutations (OS, *p* = 0.002 and DFS, *p* < 0.0001) (Figs. 3, 4). In addition, there was a progressive decline in OS but not DFS with presence of LN metastasis (*p* < 0.0001), presence of LVI (*p* = 0.049), and the presence of PNI (*p* = 0.033) (Fig. 4).

After multivariate analysis using Cox proportional hazard model, LN metastasis (*p* < 0.0001; HR = 41.5; 95% CI 7.203–239.99), and distant metastasis (*p* = 0.001; HR = 18.5; 95% CI 3.340–102.703) proved to be the only significant independent factor for OS while distant metastasis (*p* = 0.012; HR = 8.1; 95% CI 1.584–42.12) and presence of KRAS mutations (*p* = 0.008; HR = 8.7; 95% CI 1.761–43.63) were the only significant independent factors for DFS.

In KRAS mutated cases of CRC, our results demonstrated that high P4HA2 expression was the only significant poor prognostic indicator regarding DFS (*p* = 0.013) using univariate analysis (Fig. 2) as well as in multivariate analysis adjusted to age and sex (*p* = 0.028; HR = 16.1; 95% CI 1.358–192.310) (Table 4). On the other hand, presence of LN metastasis and distant metastasis were the poor prognostic factors for OS (*p* = 0.009 and *p* = 0.044) respectively.

**Table 4:** Cox regression analysis of factors affecting DFS in KRAS mutated CRC patients

Variable analysis	*p* value	HR	95% CI
P4HA2	0.028	16.159	1.358–192.310
Age	0.447	0.595	0.156–2.261
Sex	0.412	1.754	0.458–6.713

In contrast, in KRAS wild cases, the presence of LVI (*p* = 0.013) was the only independent poor prognostic factor as regards OS, while high P4HA2 expression could not be identified as an independent prognostic factor in these patients using the Cox proportional hazard model.

## Discussion

Tumor microenvironment (TME) is a dynamic process that greatly contributes to the progression of different types of cancers including CRC by promoting invasion and metastasis [4]. There are different regulators of EMT either intrinsic or extrinsic factors with P4HA2 and SLUG proteins are transcriptional factors that play an important role in the regulation of EMT [22].

To date, the role of P4HA2 and SLUG in CRC is not fully elucidated. In this study, we immunohistochemically investigated the expression of both markers in relation to different prognostic parameters in CRC and in relation to patient survival. Since KRAS mutations occur frequently in colorectal cancer, we asked whether SLUG&P4HA2 overexpression may contribute to poor prognosis in KRAS mutated CRC.

In the current study, high expression of P4HA2 was associated with bad prognostic parameters like higher T stage, presence of LN metastasis, distant metastasis, and presence of LVI and PNI. To the best of our knowledge, the prognostic role of P4HA2 was not previously investigated in CRC. However, it was studied in other tumors with similar findings [3, 7, 9]. A study by Wang et al. found that an increased level of P4HA2 mRNA was associated with advanced stage and grade in hepatocellular carcinoma [9]. In addition, a study on cervical carcinoma reported that high P4HA2 expression was correlated with advanced FIGO stage, LN metastasis, and positive LVI [3]. Also, Chang et al. demonstrated a positive relation between upregulated P4HA2 expression and metastatic potential in oral squamous cell carcinoma [7]. These concordant results highlight the critical role of P4HA2 in cancer progression.

P4HA2 is a component of prolyl 4-hydroxylase (P4H), a key enzyme in collagen biosynthesis. This enzyme catalyzes the formation of 4-hydroxyproline which is essential for the proper folding of procollagen chains [10]. In addition, P4HA2 directly modifies collagen peptides and stimulates the maturation of collagen fibers which constitute the main components of ECM [6]. Abnormal remodeling of ECM collagen is a distinctive feature of the TME and can induce EMT [6]. As collagen is the primary component of ECM fibers, several studies showed that collagen deposition within the tumor has been proven to promote tumor invasion and metastasis [6, 8, 9]. One study on glioma cells in vitro showed knockdown of P4HA2 inhibited proliferation, migration, invasion, and EMT-like phenotype of glioma cells. This sounds to consider P4HA2 as an important modulator of TME and an important target for therapy [6].

Interestingly, this study demonstrated higher expression of P4HA2 in CRC patients with associated KRAS mutations with no previous reports in CRC literature. Deng et al. reported that KRAS mutation can indirectly lead to increased expression of P4HA2 through its ability to increase NF-κB, which in turn binds to P4HA2 promotor leading to its activation [23]. This results in modulation of collagen biosynthesis and ECM changes that contribute to tumor progression.

The results of this study showed that high expression of SLUG was also associated with bad prognostic parameters; higher T stage, presence of LN metastasis, distant metastasis, and presence of LVI and PNI which is consistent with previous studies [16, 24, 25]. A study by Toiyama, et al. found higher expression of SLUG with advanced T stage, lymph node involvement, and liver metastasis in CRC patients [24]. A study by Shioiri et al. on CRC found that positive expression of SLUG was significantly associated with advanced Dukes stage and distant metastasis [16]. In addition, high expression of SLUG in gastric cancer tissue was associated with lymph node metastasis [25].

SLUG is an important transcription factor that controls different biological processes, including embryonic development, cancer progression, and stem cell reprogramming and is one of the key regulators of EMT [26]. It is implicated in repression of E-cadherin and pro-apoptotic genes, such as PTEN and p53, which facilitate tumor invasion and metastasis. Also, this makes tumor resistant to radiotherapy, chemotherapy, endocrine therapy, and even targeted therapy [27].

In addition, our study showed that higher SLUG expression was associated with KRAS mutations. This is consistent with a study by Shao et al. who found that KRAS expression strongly induced expression of mesenchymal genes including SLUG [28]. Also, a study on breast cancer reported that KRAS promotes mesenchymal phenotypes of basal-type breast cancer cells through SLUG expression among EMT transcription factors [29].

On the other hand, our result is contradictory to a previous experimental study which reported that SLUG attenuates the effects of mutant KRAS in the mouse pancreas [30].

To the best of our knowledge, this is the first study that highlights the association between P4HA2 and SLUG expression and tumor budding in CRC. In the current study, high expression of both proteins was associated with higher tumor budding.

Tumor budding has been defined as a single tumor cell or small cluster of four or fewer tumor cells at the invasive front of the tumor mass and is considered a promising and strong prognostic factor in CRC [31]. These tumor buds seem to reflect cells in a ‘hybrid’ state of EMT suggesting that these cells have a more invasive and migratory potential [32]. Since P4HA2 and SLUG are important regulators of EMT [33], we can suggest that their higher expression is correlated with higher tumor budding.

As regards survival data, the results of this study demonstrated that CRC patients whose tumors express higher levels of P4HA2 experience shorter OS and DFS, but the independent role of this high expression as a marker of poor prognosis is not proven. This result consistent with previous reports on breast cancer and cervical carcinoma [3, 8]. On the other hand, our result was contradictory with a study on pancreatic cancer which demonstrated that low expression of P4HA2 was correlated with short DFS and OS [10].

This controversy regarding the prognostic role of P4HA2 in cancer progression may be attributed to the different effects of collagen deposition in TME of several cancers. Many previous studies demonstrated that increased collagen deposition by P4HA2 offers physical and biochemical signals to enhance tumor initiation and growth. In addition, cancer cell invasion usually occurs at tumor stromal interfaces with aligned collagen fibers which facilitate cell migration and metastasis [8, 34]. On the other hand, limited studies proposed that deposition of collagen around the tumor may act as a barrier to confine tumor progression [10].

Interestingly, we first reported that high expression of P4HA2 is the only significant poor prognostic indicator in the KRAS mutated CRC group regarding DFS with no previous reports in literature. This suggests that P4HA2 may be an important indicator of recurrence in CRC patients with KRAS mutations.

In addition, this study found that CRC patients whose tumors express higher levels of SLUG experience shorter OS and DFS which is consistent with previous studies on CRC [16, 24], esophageal squamous cell carcinoma [35], and gastric carcinoma [25]. This might be explained by its ability to inhibit E-cadherin and pro-apoptotic genes which facilitate tumor invasion and metastasis, and  this makes tumor resistant to radiotherapy, chemotherapy, endocrine therapy, and even targeted therapy [27].

In this study, a strong positive correlation was present between both proteins which was not previously investigated in CRC. Although this correlation was not previously investigated in CRC, a previous study in glioma reported that expressions of SNAl1 and SLUG are regulated by P4HA2 either at transcriptional or translational level [6]. This positive correlation between two proteins may provide a novel pathway for colorectal cancer therapy.

## Conclusion

Our data highlight that both SLUG and P4HA2 expression may serve as potentially important prognostic biomarkers in CRC. In KRAS mutation group, high P4HA2 expression is the only independent prognostic factor in tumor recurrence. This may allow a better stratification of patients with high risk of recurrence in this group and may yield a novel target for diagnosis and therapy. The significant positive correlation between SLUG and P4HA2 in CRC suggests that targeting these molecules may provide a novel therapeutic strategy.

However, further molecular studies on larger sample size are recommended to confirm the prognostic role of these molecules in CRC and to investigate their possible therapeutic role especially those with KRAS mutations.

## Data Availability

The data support the findings of this study are anailable on request from the corresponding author.

## Abbreviations

AJCC: American Joint Committee on Cancer
CRC: Colorectal cancer
DFS: Disease-free survival
ECM: Extracellular matrix
EMT: Epithelial–mesenchymal transition
LN: Lymph node metastasis
LVI: Lymphovascular invasion
M: Distant metastasis
N: Lymph node metastasis
OS: Overall survival
PNI: Peri-neural invasion
T: Tumor invasion
TME: Tumor microenvironment

## References

[1] Tanaka A, Zhou Y, Shia J, Ginty F, Ogawa M, Klimstra DS, Hendrickson RC, Wang JY, Roehrl MH (2020) Prolyl 4-hydroxylase alpha 1 protein expression risk-stratifies early stage colorectal cancer. Oncotarget 11(8):813–824 32166002 10.18632/oncotarget.27491 PMC7055541

[2] Li ZN, Zhao L, Yu LF, Wei MJ (2020) BRAF and KRAS mutations in metastatic colorectal cancer: future perspectives for personalized therapy. Gastroenterol Rep (Oxf) 8(3):192–205 32665851 10.1093/gastro/goaa022 PMC7333923

[3] Cao Y, Han Q, Li J, Jia Y, Zhang R, Shi H (2020) P4HA2 contributes to cervical cancer progression via inducing epithelial-mesenchymal transition. J Cancer 11(10):2788–2799 32226497 10.7150/jca.38401 PMC7086251

[4] Bakir B, Chiarella AM, Pitarresi JR, Rustgi AK (2020) EMT, MET, plasticity, and tumor metastasis. Trends Cell Biol 30(10):764–776 32800658 10.1016/j.tcb.2020.07.003 PMC7647095

[5] Koivunen P, Salo KE, Myllyharju J, Ruddock LW (2005) Three binding sites in protein-disulfide isomerase cooperate in collagen prolyl 4-hydroxylase tetramer assembly. J Biol Chem 280(7):5227–5235 15590633 10.1074/jbc.M412480200

[6] Lin J, Jiang L, Wang X, Wei W, Song C, Cui Y, Wu X, Qiu G (2021) P4HA2 promotes epithelial-to-mesenchymal transition and glioma malignancy through the collagen-dependent PI3K/AKT pathway. J Oncol 2021:1406853 34434233 10.1155/2021/1406853 PMC8382519

[7] Chang KP, Yu JS, Chien KY, Lee CW, Liang Y, Liao CT, Yen TC, Lee LY, Huang LL, Liu SC, Chang YS, Chi LM (2011) Identification of PRDX4 and P4HA2 as metastasis-associated proteins in oral cavity squamous cell carcinoma by comparative tissue proteomics of microdissected specimens using iTRAQ technology. J Proteome Res 10(11):4935–4947 21859152 10.1021/pr200311p

[8] Xiong G, Deng L, Zhu J, Rychahou PG, Xu R (2014) Prolyl-4-hydroxylase alpha subunit 2 promotes breast cancer progression and metastasis by regulating collagen deposition. BMC Cancer 14:1 24383403 10.1186/1471-2407-14-1 PMC3880410

[9] Wang T, Fu X, Jin T, Zhang L, Liu B, Wu Y, Xu F, Wang X, Ye K, Zhang W, Ye L (2019) Aspirin targets P4HA2 through inhibiting NF-kappaB and LMCD1-AS1/let-7g to inhibit tumour growth and collagen deposition in hepatocellular carcinoma. EBioMedicine 45:168–180 31278071 10.1016/j.ebiom.2019.06.048 PMC6642319

[10] Hu D, Ansari D, Zhou Q, Sasor A, Said Hilmersson K, Andersson R (2019) Low P4HA2 and high PRTN3 expression predicts poor survival in patients with pancreatic cancer. Scand J Gastroenterol 54(2):246–251 30880498 10.1080/00365521.2019.1574360

[11] Cano A, Perez-Moreno MA, Rodrigo I, Locascio A, Blanco MJ, del Barrio MG, Portillo F, Nieto MA (2000) The transcription factor snail controls epithelial-mesenchymal transitions by repressing E-cadherin expression. Nat Cell Biol 2(2):76–83 10655586 10.1038/35000025

[12] Thiery JP, Acloque H, Huang RY, Nieto MA (2009) Epithelial-mesenchymal transitions in development and disease. Cell 139(5):871–890 19945376 10.1016/j.cell.2009.11.007

[13] Cobaleda C, Perez-Caro M, Vicente-Duenas C, Sanchez-Garcia I (2007) Function of the zinc-finger transcription factor SNAI2 in cancer and development. Annu Rev Genet 41:41–61 17550342 10.1146/annurev.genet.41.110306.130146

[14] Li Z, Mou H, Wang T, Xue J, Deng B, Qian L, Zhou Y, Gong W, Wang JM, Wu G, Zhou CF, Fang J, Le Y (2013) A non-secretory form of FAM3B promotes invasion and metastasis of human colon cancer cells by upregulating Slug expression. Cancer Lett 328(2):278–284 23059759 10.1016/j.canlet.2012.09.026 PMC7604826

[15] Come C, Magnino F, Bibeau F, De Santa BP, Becker KF, Theillet C, Savagner P (2006) Snail and slug play distinct roles during breast carcinoma progression. Clin Cancer Res 12(18):5395–5402 17000672 10.1158/1078-0432.CCR-06-0478

[16] Shioiri M, Shida T, Koda K, Oda K, Seike K, Nishimura M, Takano S, Miyazaki M (2006) Slug expression is an independent prognostic parameter for poor survival in colorectal carcinoma patients. Br J Cancer 94(12):1816–1822 16773075 10.1038/sj.bjc.6603193 PMC2361350

[17] Nagtegaal ID, Odze RD, Klimstra D, Paradis V, Rugge M, Schirmacher P, Washington KM, Carneiro F, Cree IA, Board WHOCoTE (2020) The 2019 WHO classification of tumours of the digestive system. Histopathology 76(2):182–188 31433515 10.1111/his.13975 PMC7003895

[18] Brierley JD, Gospodarowicz MK, Wittekind C (2017) TNM classification of malignant tumours. Wiley, New York 10.1016/S1470-2045(17)30438-2 PMC5851445 28677562

[19] Lugli A, Kirsch R, Ajioka Y, Bosman F, Cathomas G, Dawson H, El Zimaity H, Fléjou J-F, Hansen TP, Hartmann A (2017) Recommendations for reporting tumor budding in colorectal cancer based on the International Tumor Budding Consensus Conference (ITBCC) 2016. Mod Pathol 30(9):1299–1311 28548122 10.1038/modpathol.2017.46

[20] Toss MS, Miligy IM, Gorringe KL, AlKawaz A, Khout H, Ellis IO, Green AR, Rakha EA (2018) Prolyl-4-hydroxylase Α subunit 2 (P4HA2) expression is a predictor of poor outcome in breast ductal carcinoma in situ (DCIS). Br J Cancer 119(12):1518–1526 30410060 10.1038/s41416-018-0337-x PMC6288166

[21] Cho YA, Kim EK, Cho BC, Koh YW, Yoon SO (2019) Twist and snail/slug expression in oropharyngeal squamous cell carcinoma in correlation with lymph node metastasis. Anticancer Res 39(11):6307–6316 31704861 10.21873/anticanres.13841

[22] Pavlic A, Urh K, Stajer K, Bostjancic E, Zidar N (2021) Epithelial–mesenchymal transition in colorectal carcinoma: comparison between primary tumor, lymph node and liver metastases. Front Oncol 11:662806 34046357 10.3389/fonc.2021.662806 PMC8144630

[23] Deng Z, Zhao Z, Ning B, Basilio J, Mann K, Fu J, Gu Y, Ye Y, Wu X, Fan J, Chiao P, Hu T (2019) Nanotrap-enabled quantification of KRAS-induced peptide hydroxylation in blood for cancer early detection. Nano Res 12(6):1445–1452 10.1007/s12274-019-2405-9

[24] Toiyama Y, Yasuda H, Saigusa S, Tanaka K, Inoue Y, Goel A, Kusunoki M (2013) Increased expression of Slug and Vimentin as novel predictive biomarkers for lymph node metastasis and poor prognosis in colorectal cancer. Carcinogenesis 34(11):2548–2557 24001454 10.1093/carcin/bgt282

[25] Lee HH, Lee SH, Song KY, Na SJ, Hyun O J, Park JM, Jung ES, Choi MG, Park CH (2017) Evaluation of Slug expression is useful for predicting lymph node metastasis and survival in patients with gastric cancer. BMC Cancer 17(1):670 28974196 10.1186/s12885-017-3668-8 PMC5627408

[26] Jin B, Jin H, Wu HB, Xu JJ, Li B (2018) Long non-coding RNA SNHG15 promotes CDK14 expression via miR-486 to accelerate non-small cell lung cancer cells progression and metastasis. J Cell Physiol 233(9):7164–7172 29630731 10.1002/jcp.26543 PMC6001572

[27] Shih JY, Yang PC (2011) The EMT regulator slug and lung carcinogenesis. Carcinogenesis 32(9):1299–1304 21665887 10.1093/carcin/bgr110

[28] Shao DD, Xue W, Krall EB, Bhutkar A, Piccioni F, Wang X, Schinzel AC, Sood S, Rosenbluh J, Kim JW, Zwang Y, Roberts TM, Root DE, Jacks T, Hahn WC (2014) KRAS and YAP1 converge to regulate EMT and tumor survival. Cell 158(1):171–184 24954536 10.1016/j.cell.2014.06.004 PMC4110062

[29] Kim RK, Suh Y, Yoo KC, Cui YH, Kim H, Kim MJ, Gyu Kim I, Lee SJ (2015) Activation of KRAS promotes the mesenchymal features of basal-type breast cancer. Exp Mol Med 47(1):e137 25633745 10.1038/emm.2014.99 PMC4314588

[30] Ebine K, Chow CR, DeCant BT, Hattaway HZ, Grippo PJ, Kumar K, Munshi HG (2016) Slug inhibits pancreatic cancer initiation by blocking Kras-induced acinar-ductal metaplasia. Sci Rep 6:29133 27364947 10.1038/srep29133 PMC4929679

[31] Ozer SP, Barut SG, Ozer B, Catal O, Sit M (2019) The relationship between tumor budding and survival in colorectal carcinomas. Rev Assoc Med Bras 65(12):1442–1447 31994623 10.1590/1806-9282.65.12.1442

[32] Zlobec I, Berger MD, Lugli A (2020) Tumour budding and its clinical implications in gastrointestinal cancers. Br J Cancer 123(5):700–708 32601463 10.1038/s41416-020-0954-z PMC7462864

[33] Pal A, Barrett TF, Paolini R, Parikh A, Puram SV (2021) Partial EMT in head and neck cancer biology: a spectrum instead of a switch. Oncogene 40(32):5049–5065 34239045 10.1038/s41388-021-01868-5 PMC8934590

[34] Shields MA, Dangi-Garimella S, Krantz SB, Bentrem DJ, Munshi HG (2011) Pancreatic cancer cells respond to type I collagen by inducing snail expression to promote membrane type 1 matrix metalloproteinase-dependent collagen invasion. J Biol Chem 286(12):10495–10504 21288898 10.1074/jbc.M110.195628 PMC3060503

[35] Hasan MR, Sharma R, Saraya A, Chattopadhyay TK, DattaGupta S, Walfish PG, Chauhan SS, Ralhan R (2013) Slug is a predictor of poor prognosis in esophageal squamous cell carcinoma patients. PLoS ONE 8(12):e82846 24367561 10.1371/journal.pone.0082846 PMC3867395

